# Chemical Differentiation of Genetically Identified* Atractylodes japonica*,* A. macrocephala*, and* A. chinensis* Rhizomes Using High-Performance Liquid Chromatography with Chemometric Analysis

**DOI:** 10.1155/2018/4860371

**Published:** 2018-08-02

**Authors:** Jung-Hoon Kim, Eui-Jeong Doh, Guemsan Lee

**Affiliations:** ^1^Division of Pharmacology, School of Korean Medicine, Pusan National University, Yangsan 50612, Republic of Korea; ^2^Department of Herbology, College of Korean Medicine, Wonkwang University, Iksan 54538, Republic of Korea; ^3^Research Center of Traditional Korean Medicine, Wonkwang University, Iksan 54538, Republic of Korea

## Abstract

The rhizome of* Atractylodes japonica*, which is a herbal medicine used for gastrointestinal therapeutics, has been categorized with* A. macrocephala* rhizome or* A. chinensis* rhizome based on different therapeutic criteria in Korea, China, and Japan. In the present study, 61* A. japonica*,* A. macrocephala*, and* A. chinensis *rhizomes were collected from Korea and China and were genetically identified by internal transcribed spacer sequencing analysis. Chromatographic profiles were obtained from high-performance liquid chromatography analysis of the methanol and hot-water extracts of* Atractylodes* rhizomes and chemical differentiation of the rhizomes was carried out using chemometric statistical analyses such as principal component analysis, hierarchical clustering analysis, and Pearson's correlation coefficient analysis. The results from chromatographic profiles and chemometric analyses demonstrate that* A. japonica* rhizomes showed apparent chemical differences from* A. macrocephala* and* A. chinensis* rhizomes in the methanol extracts. In contrast, no clear distinction was apparent for the hot-water extracts of* Atractylodes* rhizomes, especially* A. chinensis* rhizomes. These results indicate that there is a clear chemical difference between* A. japonica* and* A. macrocephala* rhizomes; however, the chemical diversity of* A. chinensis* rhizome shows different chemical relationships with* A. japonica* or* A. macrocephala* rhizome, dependent on the chemical features.

## 1. Introduction

The rhizomes of* Atractylodes japonica*,* A. macrocephala*, and* A. chinensis* (Asteraceae) have been therapeutically used to treat gastrointestinal disorders in Korea, China, and Japan. The medicinal classification of these rhizomes differs in the pharmacopeia of each country: the rhizomes of* A. japonica* and* A. macrocephala* are categorized together in Korea, while the rhizome of* A. japonica* is unofficial in the pharmacopeia but is categorized with that of* A. chinensis* in China [1–3]. This inconsistency, especially with respect to* A. japonica* rhizome, causes confusion in the use of the* Atractylodes* rhizomes between countries with different categorization of such medicinal herbs.

For the criteria of the use of Atractylodes rhizomes, we reported in our previous study that the rhizome of* A. japonica* showed a closer genetic relationship and higher chemical similarity with* A. chinensis* rhizome than with* A. macrocephala* rhizome, and more consistent therapeutic effects would be expected when similarly categorized. Therefore, we proposed that* A. japonica* rhizome is genetically and chemically distinct from* A. macrocephala* rhizome and that the former should be used under the same medicinal categorization as* A. chinensis* rhizome [4]. However, there were a number of limitations at study, such as insufficient sample numbers, a lack of detailed sample information, and single solvent extraction. Hence, genetic and chemical relationships among* Atractylodes* rhizomes should be evaluated under improved conditions.

In the present study, a total of 61 Atractylodes samples were collected and their original species were identified using internal transcribed spacer (ITS) sequences from nuclear ribosomal DNA (nrDNA), which is the identification method mostly used for genetic confirmation of plant species [5–7]. Genetically identified samples were extracted with either organic or aqueous solvents, and the extracts were analyzed using a high-performance liquid chromatography-diode array detector (HPLC-DAD) instrument to compare their chromatographic profiles [8]. The chemical relevance of the rhizomes of* A. japonica*,* A. macrocephala*, and* A. chinensis* was determined based on principal component analysis (PCA), hierarchical clustering analysis (HCA), and Pearson's correlation analysis.

## 2. Materials and Methods

### 2.1. Plant Materials and Reagents

Methanol, water, and acetonitrile (HPLC grade) were purchased from J.T.Baker (Phillipsburg, NJ, USA). Trifluoroacetic acid (TFA) was purchased from Sigma-Aldrich (St Louis, MO, USA). Forty-one samples of* A. japonica* rhizomes, 11 samples of* A. macrocephala* rhizomes, and 9 samples of* A. chinensis* rhizomes were obtained from the wild, agricultural fields, or local herbal markets in Korea and China in 2016. The samples were authenticated by their morphological characteristics and by ITS sequencing analysis. The samples were coded as AJ for* A. japonica *rhizomes, AM for* A. macrocephala *rhizomes, and AC for* A. chinensis* rhizomes (Table 1). Voucher specimens have been deposited at the School of Korean Medicine, Pusan National University.

### 2.2. Preparation of Genomic DNA

Genomic DNA was extracted from the crude drugs of Atractylodes rhizomes according to the modified manuals of NucleoSpin® Plant II kit (Macherey-Nagel, Dueren, Germany). For some samples, 10% cetyltrimethyl ammonium bromide and 0.7 M NaCl were used to remove the phenolic compounds and polysaccharides.

### 2.3. PCR Amplification

For ITS amplification, PCR was performed using a T-personal cycler (Biometra; Göttingen, Germany). Briefly, 600 nM of the primer set of ITS1 (5′-TCCGTAGGTGAACCTGCGG-3′) and ITS4 (5′-TCCTCCGCTTATTGATATGC-3′) [9], AccuPower® GoldHotstart Taq PCR PreMix (Bioneer, Daejeon, Korea), and 50 ng of genomic DNA were used for PCR amplification. PCR cycling conditions, which were followed by predenaturation process (95°C, 5 min), were as follows: denaturation process (95°C, 30 s); annealing progress (52°C, 30 s); extension process (72°C, 40 s) × 36 cycles; final extension process (72°C, 5 min). The amplified PCR product was separated from other gradients by using 1.5% agarose gel electrophoresis after staining by the addition of SafeView™ (abm; Vancouver, Canada). Amplified products were analyzed using MyImage (Seoulin Biotechnology; Seongnam, Gyeonggi-do, Korea).

### 2.4. Determination of DNA Sequence of PCR Product

PCR product separated from agarose gel was cloned by using a TOPcloner™ TA-Blunt kit (Enzynomics, Daejeon, Korea) and the DNA sequence of the cloned PCR product was determined after interpretation performed by Bioneer (Daejeon, Korea).

### 2.5. Analysis of DNA Sequence and Preparation of Dendrogram

DNA sequences were analyzed using the ClustalW multiple sequence alignment (BioEdit, v7.0.9; available at http://www.mbio.ncsu.edu/BioEdit/page2.html) and the phylogenetic tree was created using DNADist (BioEdit). To study the relationships among* Atractylodes* rhizomes, the nucleotide sequences of the genera Atractylis and Carlina deposited in NCBI GenBank were used. The genera Brachylaena, Cardopatium, Cirsium, Echinops, Phonus, and Tarchonanthus were used as outgroups in the phylogenetic analyses, based on previous studies [10, 11]. ITS sequences of these taxa were collected from NCBI GenBank. The ITS sequences of nine specimens (coded with Arabic numerals) of* Atractylodes* plants were collected as species reference samples for identification of* Atractylodes* rhizomes in previous research [4], and these were used as a reference for species.

### 2.6. Preparation of Samples for HPLC Analysis

For the methanol extract, dried powder of the rhizomes (1.0 g) was extracted with 10 mL of methanol using an ultrasonic extractor (Power Sonic 520; Hwashin Tech, Daegu, Korea) for 30 min. The extract was centrifuged at 10,000 rpm for 5 min. The supernatant was transferred to a 1.5 mL microtube and evaporated using a nitrogen gas blowing concentrator (MGS–2200; Eyela, Miyagi, Japan). Residue was dissolved in methanol to a concentration of 10,000 *μ*g/mL and the extract solution was filtered through a 0.2 *μ*m syringe filter (BioFact; Daejeon, Korea) prior to HPLC injection.

For the hot-water extract, dried powder of the rhizome (0.5 g) was extracted in a 100 mL glass bottle with 20 mL of distilled water using a microwave oven (BP-111-RS; Microwave Research & Applications, Inc., IL, USA) at 90°C for 20 min. The water extract was transferred to a 15 mL conical tube and centrifuged at 3000 rpm for 10 min. The supernatant was lyophilized using a freeze-dryer (IlShinBioBase; Dongducheon, Gyeonggi-do, Korea). The powder of dried extract (20 mg) was dissolved in 1 mL of HPLC grade water and then the solution was centrifuged at 10,000 rpm for 5 min. The supernatant was filtered through a 0.2 *μ*m syringe filter (BioFact) prior to HPLC injection.

### 2.7. HPLC Conditions for Chromatographic Fingerprinting

An Agilent 1260 liquid chromatography system (Agilent Technologies; Palo Alto, CA, USA) equipped with an autosampler, degasser, quaternary solvent pump, and diode array detector (DAD) was used for chromatographic fingerprinting. The data were processed with Chemstation software (Agilent Technologies). The separation of compounds was carried out on a Capcell Pak Mg II C_18_ column (4.6 mm × 250 mm, 5 *μ*m; Shiseido, Tokyo, Japan) at 35°C. The flow rate was 1 mL/min and the injection volume was 10 *μ*L.

For analysis of the methanol extract, the mobile phase consisted of water (A) and acetonitrile (B), with the following gradient elution: 55% (B) over 0–1 min, 55–60% (B) over 1–35 min, 60% (B) over 35–36 min, 60–90% (B) over 36–51 min, 90% (B) over 51–52 min, and then reequilibration to 55% (B) until the end of the analysis. Detection was performed with a UV detector at wavelengths of 230, 255, 275, 315, and 340 nm.

For analysis of the hot-water extract, the mobile phase consisted of 0.1% TFA in water (A) and acetonitrile (B), with the following gradient elution: 5% (B) over 0–5 min, 5–40% (B) over 5–35 min, 40% (B) over 35–36 min, 40–70% (B) over 36–51 min, 70% (B) over 51–52 min, and then reequilibration to 5% (B) until the end of the analysis. Detection was performed with a UV detector at wavelengths of 225, 255, 275, 295, and 325 nm.

### 2.8. Chemometric Statistical Analysis

The 61 samples that were genetically identified and recoded were used for PCA, HCA, and Pearson's correlation analysis. In total, 45 and 31 peaks were selected as reference peaks (> 1.0% of total peak area) for the methanol extract and water extract, respectively, at their optimal UV absorption, and their absolute areas were calculated by peak area integration for chromatographic fingerprinting. A matrix composed of the rows (Atractylodes sample) and columns (absolute area of each reference peak) was used to construct the PC plot and dendrogram and for Pearson's correlation analysis, using the open source software R (v. 3.4.3; The R Foundation for Statistical Computing).

## 3. Results

### 3.1. ITS Genotype and Genetic Identification of Atractylodes Rhizomes

Amplification of the ITS region produced overall 733 bp of nucleotide sequences from 61 samples listed in Table 1 and from nine dried voucher specimens [4]. The ITS sequences of samples were determined by comparing DNA sequences registered in NCBI GenBank as well as in [12], with the following accession numbers: for* A. japonica* AB219405, for* A. macrocephala* AB219406, for* A. lancea* AB219407, for* A. chinensis* AB219408, and for* A. koreana* AB219409. As shown in previous research [4], nucleotide substitutions were observed in 37 sites on the ITS regions of Atractylodes samples. Type 1, the ITS sequence of* A. japonica*, showed multiple sequences compared with the other species; Types 2 and 3 were the genotypes of* A. macrocephala* and* A. lancea*, respectively. Type 4, the genotype of* A. chinensis*, was identical to Type 5, the genotype of* A. koreana*.

All 11 samples labeled AM, were determined as* A. macrocephala*; no difference of DNA sequence between samples was observed. Among the nine samples labeled AC, determined as* A. chinensis*, AC4, AC7, and AC8 showed differences in nucleotide sequence. The sequence of AC4, AC7, and AC8 differed from that of Type 4 by 1 bp (A → G) at nucleotide position 128 bp, which indicates intraspecific variation. All 41 samples labeled AJ were determined as* A. japonica*. Unlike in other species, some intraspecific variation was observed among the 41 AJ samples. Most of the differences were multisequences, which have already been shown to be Type 1 [4].

### 3.2. Genetic Relationship of the Atractylodes Rhizomes

**Figure 1:**
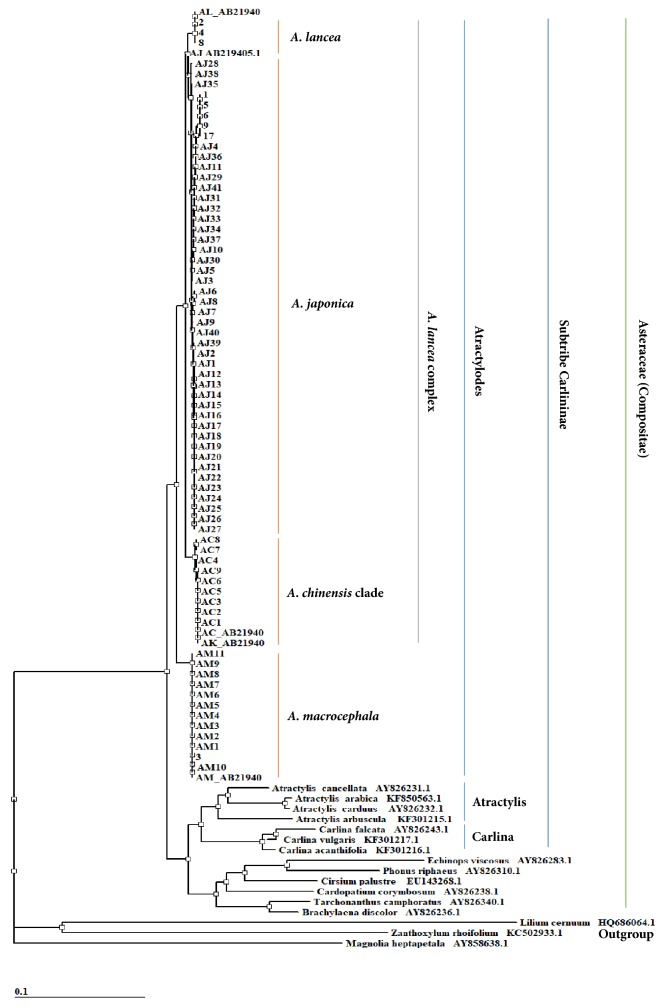
Phylogenic tree from DNADist (neighbor phylogenetic tree) analysis of the ITS nucleotide sequences. The ITS sequences of taxa with Atractylis, Carlina, and Outgroup were downloaded from NCBI GenBank. The samples with Arabic numerals were dried voucher specimens deposited at the Korea Institute of Oriental Medicine.

Sixty-one samples of Atractylodes rhizomes were identified as* A. chinensis*,* A. japonica*, and* A. macrocephala*. Phylogenetic classifications based on the ITS region were made and the inferred evolutionary relationships among* Atractylodes* rhizomes are represented as a phylogenetic tree. The genus* Atractylodes* is well separated from other close genera and outgroups. The samples of* A. japonica*,* A. lancea*, and* A. chinensis* formed* A. lancea* complex, whereas those of* A. macrocephala* formed their own* A. macrocephala* complex (Figure 1).

### 3.3. Chromatographic Profiling of the Methanol and Hot-Water Extracts of Atractylodes Samples

**Figure 2:**
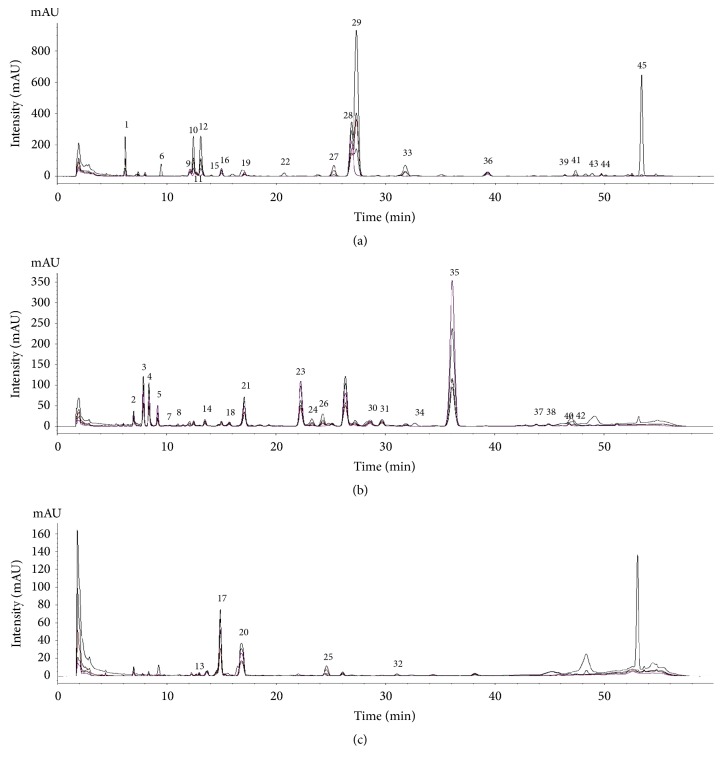
Representative chromatograms of methanol extracts of* Atractylodes japonica* (AJ1, (a)),* A. chinensis* (AC1, (b)), and* A. macrocephala* (AM1, (c)) at UV wavelengths of 230, 255, 275, 315, and 340 nm.

**Figure 3:**
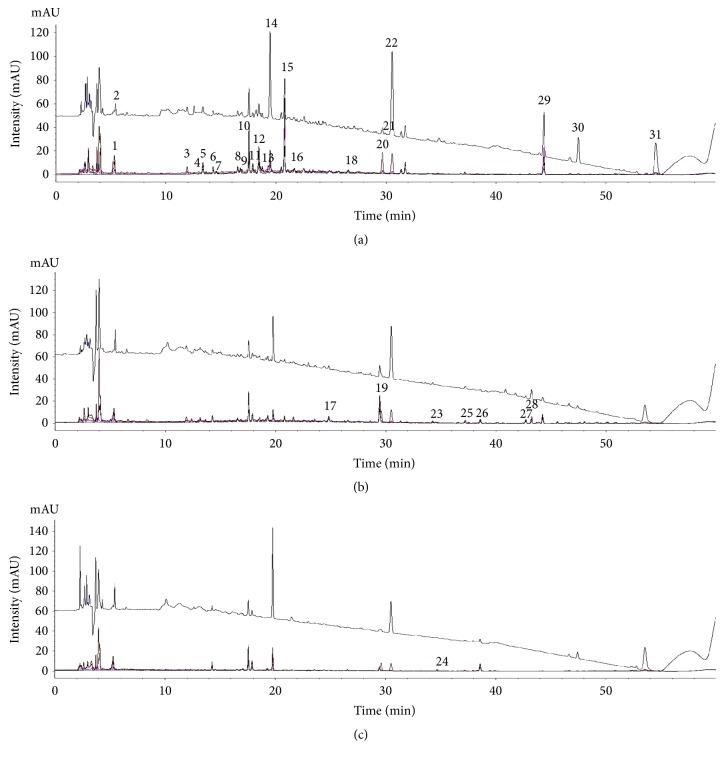
Representative chromatograms of hot-water extracts of* A. japonica* (AJ1, (a)),* A. chinensis* (AC1, (b)), and* A. macrocephala* (AM1, (c)) at UV wavelengths of 225, 255, 275, 295, and 325 nm.

**Figure 4:**
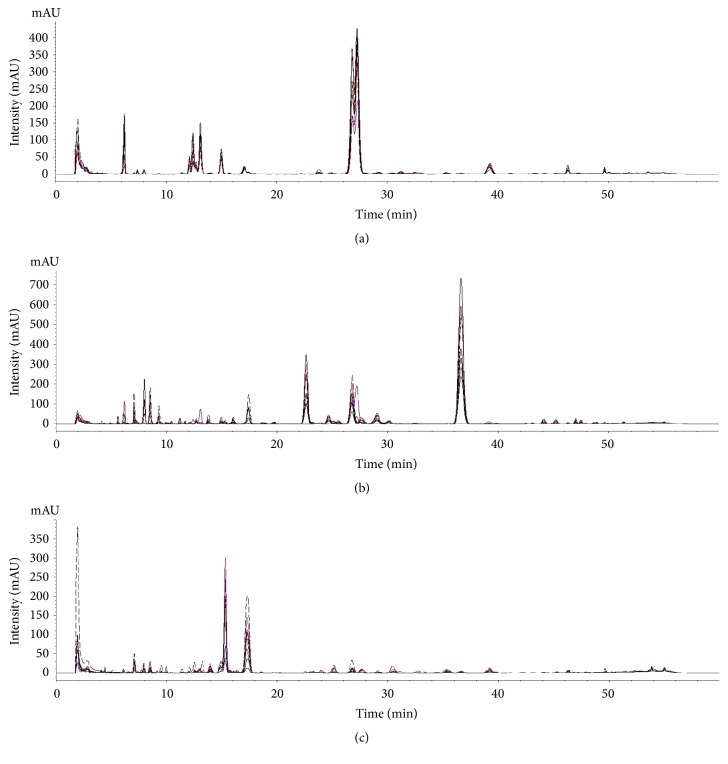
Representative chromatograms of methanol extracts of* A. japonica* (AJ1–9, (a)),* A. chinensis* (AC1–9, (b)), and* A. macrocephala* (AM1–9, (c)) at UV wavelength of 315 nm.

**Figure 5:**
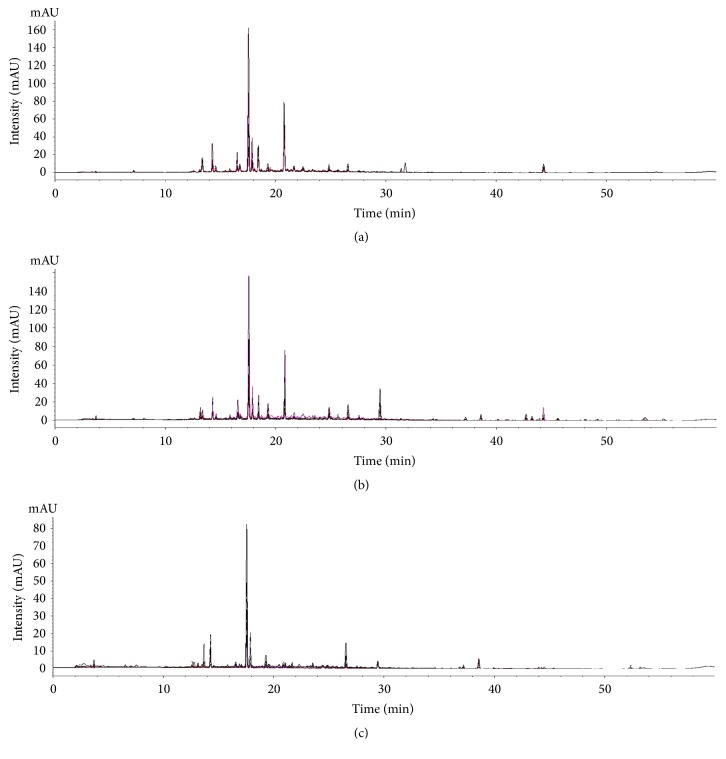
Representative chromatograms of hot-water extracts of* A. japonica* (AJ1–9, (a)),* A. chinensis* (AC1–9, (b)), and* A. macrocephala* (AM1–9, (c)) at UV wavelength of 325 nm.

Chromatographic profiles of representative samples (AJ1, AC1, and AM1) at all UV wavelengths were compared; a total of 45 peaks and 31 peaks for the methanol and water extracts, respectively, were selected for comparison by macroscopic observation and further chemometric analysis (Figures 2 and 3 and Table S1). There were distinct differences in chromatographic patterns of the methanol extracts of AJ, AC, and AM, and 28 out of 45 reference peaks were shared among the methanol extracts of* Atractylodes *samples (Figure 4). In contrast, chromatographic differences among the water extracts were less distinctive, with 18 out of 31 references peaks being shared among the chromatograms from the hot-water extracts (Figure 5).

### 3.4. Clustering Analysis of Atractylodes Samples Using Chemometric Statistical Methods

Chromatographic profiles of the methanol and water extracts from Atractylodes samples were further analyzed by principal component analysis (PCA), hierarchical clustering analysis (HCA), and of Pearson's correlation coefficient to estimate the relationship between the* Atractylodes* samples.

**Figure 6:**
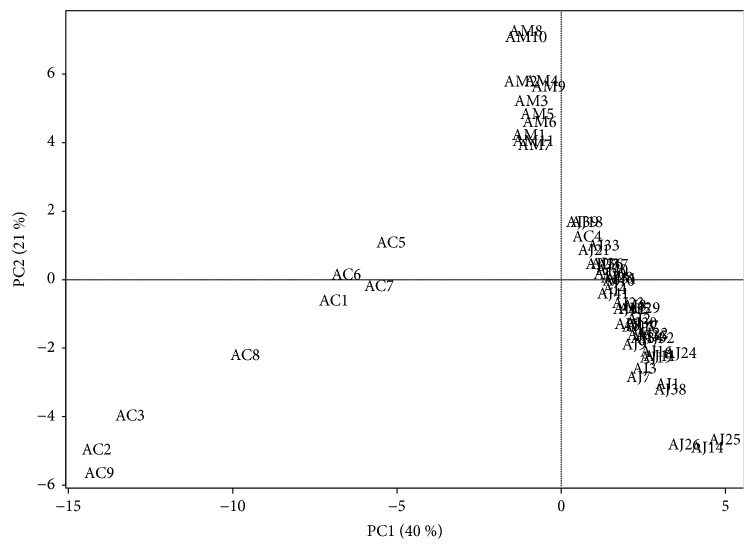
Score plot of principal components (PC1 versus PC2) on the variables (absolute area of reference peaks) with* Atractylodes* samples from the methanol extracts. PC1 and PC2 represent 40% and 21% of the total variance, respectively. AC:* A. chinensis* Koidz.; AJ:* A. japonica* Koidz.; AM:* A. macrocephala* Koidz.

**Figure 7:**
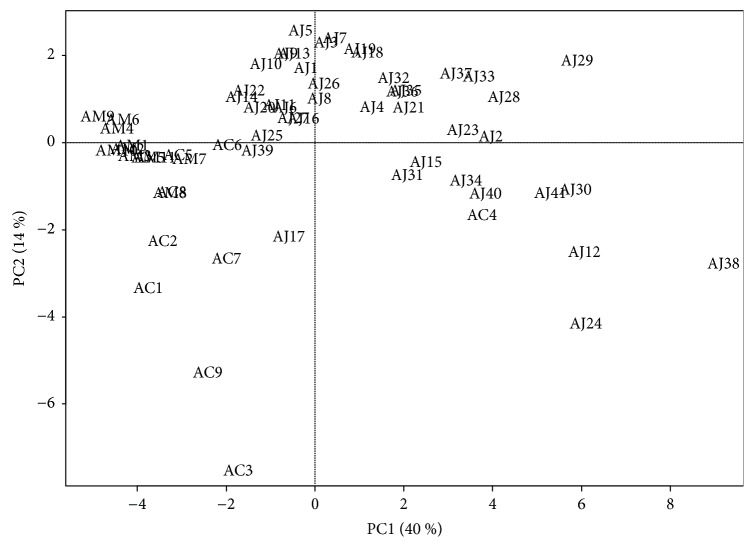
Score plot of principal components (PC1 versus PC2) on the variables (absolute area of reference peaks) with* Atractylodes* samples from the hot-water extracts. PC1 and PC2 represent 40% and 14% of the total variance, respectively. AC:* A. chinensis* Koidz.; AJ:* A. japonica* Koidz.; AM:* A. macrocephala* Koidz.

In the principal component (PC) plot of the methanol extracts of the samples, three distinct clusters were observed: an AJ group (AJ1–41 including AC4), an AM group (AM1–10), and an AC group (AC1–9 except AC4). The distribution of PC1 score showed that the samples in the AJ group were plotted closer to those in the AM group than those in the AC group; in contrast, in the PC2 score, the samples in the AC group were located closer to those in the AJ group than those in the AM group (Figure 6). However, the PC plot of the hot-water extracts of the samples differed from that of the methanol extracts. As the samples were distributed in a wide range of PC1 and PC2 scores, no distinct clusters were observed. AC4 and AC6 were plotted closer to the location of AJ samples, whereas AC5 and AC8 overlapped into the plots of AM samples (Figure 7).

**Figure 8:**
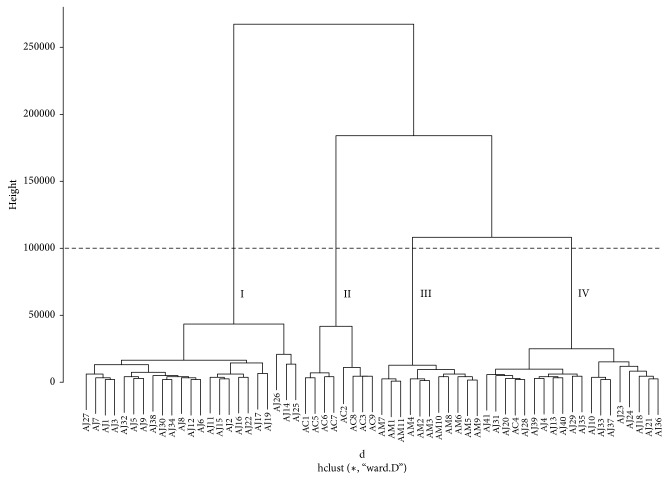
Hierarchical clustering analysis of Atractylodes samples from methanol extracts. AC:* A. chinensis* Koidz.; AJ:* A. japonica* Koidz.; AM:* A. macrocephala* Koidz.

**Figure 9:**
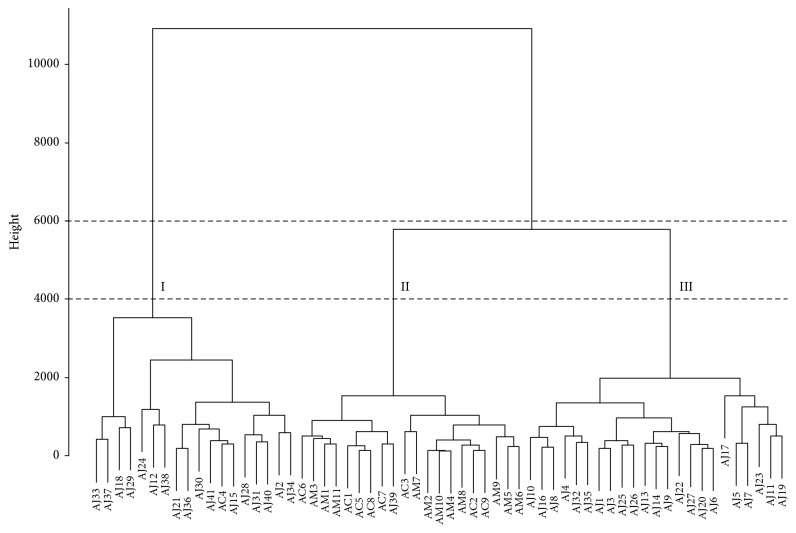
Hierarchical clustering analysis of Atractylodes samples from hot-water extracts. AC:* A. chinensis* Koidz.; AJ:* A. japonica* Koidz.; AM:* A. macrocephala* Koidz.

The dendrogram of methanol extracts from HCA showed similar groups as obtained from PCA; namely, AJ, AC, and AM samples formed their own groups (groups II, III, and IV) except for AC4 in the AJ group. However, several AJ samples were divided into a separate group below a height of 250000 and clustered in group I below a height of 100000 (Figure 8). For the dendrogram of the methanol extracts, the water extracts of AJ samples were also divided into two distinct groups (groups I and II), whereas those of AC and AM samples were not clearly distinguished within their own groups because they were not separated in the subgroup of group II, below a height of 4000 (Figure 9).

### 3.5. Evaluation of the Correlation between A. japonica, A. chinensis, and A. macrocephala Samples by Pearson's Correlation Coefficient

**Figure 10:**
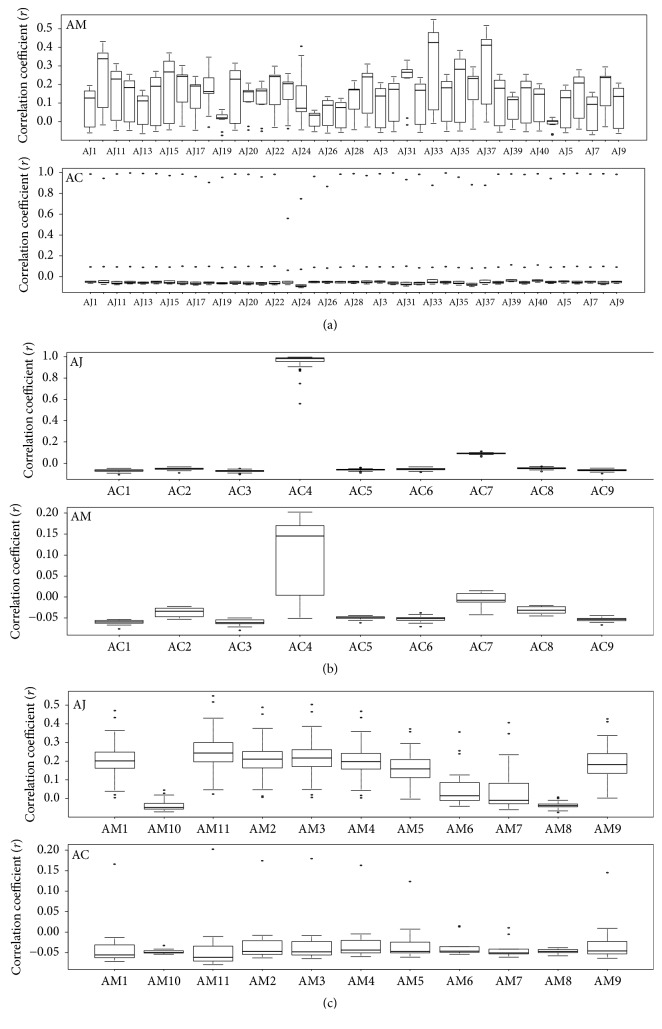
Average coefficients of Pearson's correlation coefficient of* Atractylodes* samples from methanol extracts. (a) AJ–AC and AJ–AM, (b) AC–AJ and AC–AM, and (c) AM–AJ and AM–AC. AC:* A. chinensis *Koidz.; AJ:* A. japonica* Koidz.; AM:* A. macrocephala* Koidz.

In the methanol extracts, the mean value of the Pearson's correlation coefficient (*r*) of each AJ sample ranged from 0.01 to 0.31 (except for AJ41;* r* = –0.01) for the correlation of AM samples and from 0.01 to 0.1 for the correlation of AC samples (Figure 10(a)). The mean correlation coefficient of each AC sample ranged from –0.08 to 0.1 (except for AC4;* r* = 0.95) for the correlation of AJ samples and from –0.07 to 0.1 for the correlation of AM samples (Figure 10(b)). The mean correlation coefficient of each AM sample ranged from –0.05 to –0.01 for the correlation of AC samples and from –0.03 to 0.22 for the correlation of AJ samples (Figure 10(c)).

**Figure 11:**
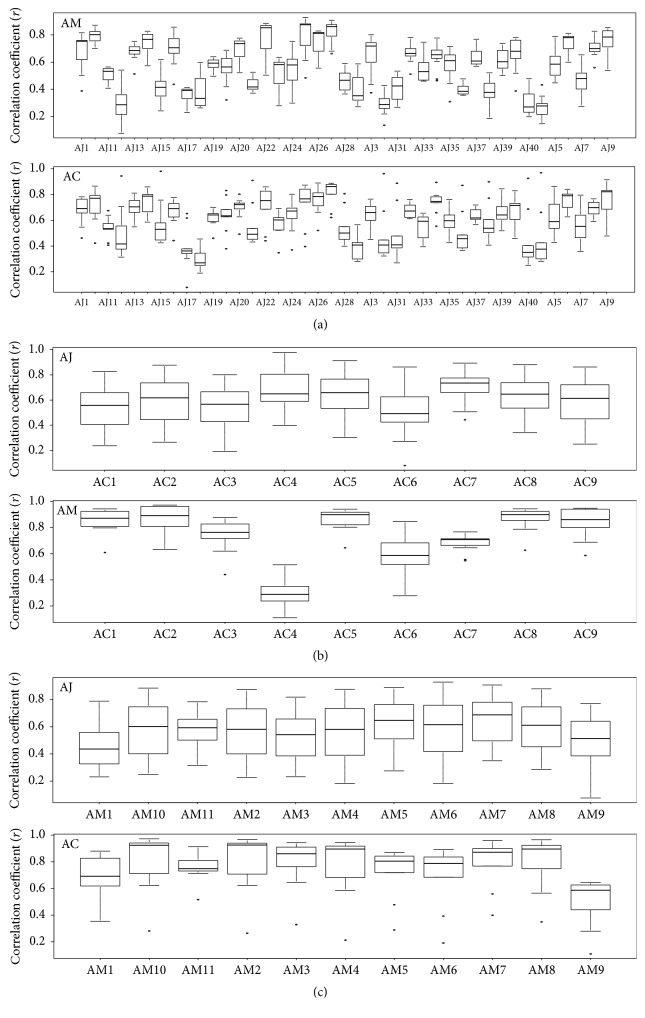
Average coefficients of Pearson's correlation coefficient of* Atractylodes* samples from hot-water extracts. (a) AJ–AC and AJ–AM, (b) AC–AJ and AC–AM, and (c) AM–AJ and AM–AC. AC:* A. chinensis *Koidz.; AJ:* A. japonica* Koidz.; AM:* A. macrocephala* Koidz.

In the hot-water extracts, the mean correlation coefficient of each AJ sample ranged from 0.25 to 0.80 for the correlation of AM samples and from 0.30 to 0.81 for the correlation of AC samples (Figure 11(a)). The mean correlation coefficient of each AC sample ranged from 0.55 to 0.72 for the correlation of AJ samples and from 0.29 to 0.87 for the correlation of AM samples (Figure 11(b)). The mean correlation coefficient of each AM sample ranged from 0.49 to 0.79 for the correlation of AC samples and from 0.45 to 0.65 for the correlation of AJ samples (Figure 11(c)). The mean and median values of Pearson's correlation coefficient from* Atractylodes* samples are shown in Table 2.

## 4. Discussion

We previously proposed that the rhizome of* A. japonica* (AJ) should be considered for medicinal use as being in the same medicinal category as that of* A. chinensis* (AC) because of their genetic and chemical similarity to that of* A. macrocephala* (AM) [4]. However, an unresolved issue is whether the rhizome of* A. japonica* should be medicinally used in parallel with the rhizome of* A. macrocephala* or with the rhizome of* A. chinensis*. Therefore, building on our previous work, in this study, 61 Atractylodes rhizomes that were collected from Korea and China in 2016 were genetically identified by their original species and their chemical differentiation was carried out by chromatographic profiling and chemometric statistical analysis.

In the methanol extracts, three apparent clusters of* Atractylodes* samples in the PC plot (AJ samples + AC4, AC samples, and AM samples) showed different proximity among each group. PC1 and PC2 scores, the most and next most influential factors of clustering in the PC plot, provided the group of AJ samples with more proximity to the group of AM samples than that of AC samples which results in better chemical relevance, as the PC scores of the AJ samples were closer to those of AM samples [13, 14]. The proximity among the groups of AJ, AC, and AM samples was also confirmed by the dendrogram from HCA, which produces clusters to classify samples by measuring the distance between samples [15, 16]. The dendrogram also indicated apparent groups of AJ, AC, and AM samples, and samples in the AJ group (cluster IV) showed closer chemical similarity to those in the AM group (cluster III) than to those in the AC group (cluster II) below a height of 100000, as samples with a similar distance were involved in the same group [17].

Pearson's correlation coefficient (*r*) analysis among the samples also supports the clusters obtained from PCA and HCA. The overall correlation between different* Atractylodes* species was not strong and was even negative, indicating that the samples of AJ, AM, and AC groups represented a weak correlation and, hence, were separated from each other [18, 19]. Stronger correlation was observed between samples from the AJ–AM group, with positive and higher *r* values, than between samples from the AJ–AC group, with negative and lower *r* values (close to 0) [20, 21].

In the hot-water extracts, the clusters of the samples according to their original species were not distinct in the PC plot, in which the samples from the same species were mostly gathered together. The AJ samples exhibited higher chemical similarity to a number of AC samples from the most influential PC1 scores, whereas the PC2 scores of AJ samples indicated a closer relationship to AM samples. Rather, AC and AM samples showed higher proximity to each other. These relationships between samples were also observed in the dendrogram: the AJ samples formed separate groups, whereas the AC and AM samples were consolidated in subgroups of cluster II, which makes the apparent delineation between AC and AM samples more difficult, as evaluated by the higher *r* values between two species. Moreover, AJ samples indicated a stronger correlation with AC samples than with AM samples overall.

Unlike the distribution of the samples in the PC plot, about half of the AJ samples formed a separate AJ group (each cluster I in the dendrograms from the methanol and water extracts, respectively) in the dendrogram from HCA, showing least similarity with AC, AM, and the remaining AJ samples. In contrast, but similar to the results from PCA, each AJ sample was strongly correlated with other AJ samples from the methanol and hot-water extracts, with an *r* value close to 1 [19] (Supplementary Figures S1 and S2). These results might be ascribed to the differences in mathematical and statistical methods between HCA and PCA [22–24].

In contrast to our previous study [4], the chemometric results of the present study demonstrate that the chemical correlation and relevance of AJ samples, particularly in the methanol extracts (organic extract), were weaker with AC samples than with AM samples, although AJ samples have a closer genetic relationship to AC samples than to AM samples. Regarding these results, an important difference from the previous work that should be considered, however, is that there was always a distance between AJ and AM samples in the previous and present study, which means that AJ samples were not chemically analogous to AM samples; that is, they had their own clusters of samples. Another point is that the location of AC samples changed from “inside” to “outside” the cluster of AJ samples, indicating that AC samples in the present work showed less chemical homogeneity to AJ samples, as reported previously [25, 26]. This might be explained by the fact that geographical diversity can lead to a wide range of variation in the content of chemical components from the rhizome of* A. chinensis* [27, 28]. This is because environmental factors, such as climate, temperature, humidity, soil, and altitude, and the corresponding adaptation of plants are crucial for the biosynthesis and production of chemical components and secondary metabolites [29, 30], and such environmental differences can also influence the chemical variation in the plants of genus* Atractylodes* [31–34].

Based on the results from chromatographic profiles and chemometric analyses,* A. japonica* rhizomes could apparently be differentiated from* A. macrocephala* and* A. chinensis* rhizomes by their methanol-soluble components, whereas* A. chinensis* rhizomes were not clearly distinguishable from* A. japonica* and* A. macrocephala* rhizomes by their hot-water-soluble components. These results indicate that the chemical difference between* A. japonica* and* A. macrocephala* was distinct in both extracts, as also reported previously [4]. However, the difference between* A. japonica* and* A. chinensis* was dependent on the chemical features that were identified despite their closer genetic relationship. Moreover, aqueous components from* A. chinensis* rhizome were chemically closer to those from* A. macrocephala* rhizome, although they are categorized as different therapeutic agents. Further pharmacological and clinical evidence is necessary to confirm the chemical correlation between* Atractylodes* rhizomes.

## 5. Conclusion

In this study, 61 Atractylodes rhizomes that were collected from Korea and China in 2016 were genetically identified by their original species by ITS DNA sequencing analysis:* A. japonica*,* A. macrocephala*, and* A. chinensis*. Chemical differentiation was carried out by chromatographic profiling and chemometric statistical analysis, namely, PCA, HCA, and Pearson's correlation analysis, using the methanol and hot-water extracts of* Atractylodes* samples. The results from chemical fingerprinting and statistical analyses demonstrated that* A. japonica* rhizomes were chemically distinct from* A. macrocephala* rhizomes. However,* A. chinensis* rhizomes represented diverse chemical variation showing a wide range of relationships to* A. japonica* and* A. macrocephala *rhizomes, presumably arising from their environmental differences.

## Figures and Tables

**Table 1:** Genetically original species and collecting area of Atractylodes samples.

Code	Location	Country	Date of collection	Code	Location	Country	Date of collection
AJ1	Euiseong, Gyeongbuk	Korea	Jan, 2016	AJ32	Yeongdeok, Gyeongbuk	Korea	Feb, 2016
AJ2	–	Korea	Feb, 2016	AJ33	Yeongcheon, Gyeongbuk	Korea	Feb, 2016
AJ3	Euiseong, Gyeongbuk	Korea	Feb, 2016	AJ34	Yangpyeong, Gyeonggi	Korea	Feb, 2016
AJ4	–	Korea	Jan, 2016	AJ35	Hongcheon, Gangwon	Korea	Feb, 2016
AJ5	Yeongdeok, Gyeongbuk	Korea	Feb, 2016	AJ36	Andong, Gyeongbuk	Korea	Feb, 2016
AJ6	–	Korea	Jan, 2016	AJ37	Yeongcheon, Gyeongbuk	Korea	Feb, 2016
AJ7	Bonghwa, Gyeongbuk	Korea	Feb, 2016	AJ38	Cheongsong, Gyeongbuk	Korea	Feb, 2016
AJ8	Yeongcheon, Gyeongbuk	Korea	Feb, 2016	AJ39	Neimengu	China	Apr, 2016
AJ9	Yeongcheon, Gyeongbuk	Korea	Feb, 2016	AJ40	Hebei	China	Jul, 2016
AJ10	–	Korea	Mar, 2016	AJ41	–	China	Aug, 2016
AJ11	–	China	Jan, 2016	AM1	–	China	Jan, 2016
AJ12	–	China	Jan, 2016	AM2	Anhui	China	Jul, 2016
AJ13	Yeongwol, Gangwon	Korea	Feb, 2016	AM3	–	China	Jan, 2016
AJ14	Hwasun, Jeonnam	Korea	Jan, 2016	AM4	Zhejiang	China	Mar, 2016
AJ15	Heilongjiang	China	Mar, 2016	AM5	Zhejiang	China	Feb, 2014
AJ16	–	Korea (North)	Jan, 2016	AM6	–	China	Jan, 2016
AJ17	–	Korea	Jan, 2016	AM7	–	China	Jan, 2016
AJ18	Yeongdeok, Gyeongbuk	Korea	Jan, 2016	AM8	Shanghai	China	Jul, 2016
AJ19	–	Korea	Jan, 2016	AM9	Panan, Zhejiang	China	Jul, 2016
AJ20	–	China	Jan, 2016	AM10	Panan, Zhejiang	China	Jul, 2016
AJ21	–	China	Jan, 2016	AM11	–	China	Aug, 2016
AJ22	Heilongjiang	China	Jan, 2016	AC1	Hubei	China	Mar, 2016
AJ23	Suwon, Gyeonggi	Korea	Jan, 2016	AC2	–	China	Jan, 2016
AJ24	Jangsu, Jeonbuk	Korea	Jan, 2016	AC3	Chengde, Hebei	China	Jul, 2016
AJ25	Yangsan, Gyeongnam	Korea	Oct, 2016	AC4	–	China	Jan, 2016
AJ26	Cheongyang, Chungnam	Korea	May, 2016	AC5	Chengde, Hebei	China	Jul, 2016
AJ27	Heilongjiang	China	Aug, 2016	AC6	Hebei	China	Jul, 2016
AJ28	Yeongcheon, Gyeongbuk	Korea	Feb, 2016	AC7	Neimengu	China	Mar, 2014
AJ29	Yeongcheon, Gyeongbuk	Korea	Feb, 2016	AC8	–	China	Jan, 2016
AJ30	–	Korea	Feb, 2016	AC9	–	China	Jan, 2016
AJ31	Jecheon, Chungbuk	Korea	Feb, 2016				

AJ, *Atractylodes japonica*; AM, *A. macrocephala*; AC, *A. chinensis*. “–”: unclear location.

**Table 2:** Mean and median value of Pearson's correlation coefficient (*r*) among AJ, AM, and AC samples.

Extraction	Sample	Parameter	Correlation coefficient (*r*)		
			AJ	AM	AC
Methanol extract	AJ	Mean	–	0.131	0.069
		Median	–	0.146	–0.054
	AM	Mean	0.131	–	–0.028
		Median	0.146	–	–0.048
	AC	Mean	0.069	–0.028	–
		Median	–0.054	–0.048	–

Water extract	AJ	Mean	–	0.567	0.609
		Median	–	0.584	0.626
	AM	Mean	0.567	–	0.733
		Median	0.584	–	0.800
	AC	Mean	0.609	0.733	–
		Median	0.626	0.800	–

AJ, *A. japonica*; AM, *A. macrocephala*; AC, *A. chinensis*.

## Data Availability

The data used to support the findings of this study are available from the corresponding author upon request.

## Supplementary Materials

Supplementary Materials To read our article more intuitively, supplementary material is added as Table S1 and Figures S1 and S2. Table S1: peak number, retention time, relative retention time, and UV absorption wavelength of the reference peak in the chromatograms of the methanol extract and the hot-water extract. Figure S1: average coefficients of Pearson's correlation coefficient of* Atractylodes* samples from methanol extracts (AJ–AJ, AC–AC, and AM–AM). AC:* A. chinensis *Koidz.; AJ:* A. japonica* Koidz.; AM:* A. macrocephala* Koidz. Figure S2: average coefficients of Pearson's correlation coefficient of* Atractylodes* samples from hot-water extracts (AJ–AJ, AC–AC, and AM–AM). AC:* A. chinensis *Koidz.; AJ:* A. japonica* Koidz.; AM:* A. macrocephala* Koidz.
