# Supplementary material for: Chemical Differentiation of Genetically Identified Atractylodes japonica, A. macrocephala, and A. chinensis Rhizomes Using High-Performance Liquid Chromatography with Chemometric Analysis
Source: Evid Based Complement Alternat Med. 2018 Aug 2;2018:4860371. doi: 10.1155/2018/4860371 (PMC6098908; doi:10.1155/2018/4860371)

Table S1.

TABLE S1: Peak number, retention time, relative retention time, and UV absorption wavelength of the reference peak in the chromatograms of the methanol extract and the hot-water extract

| Methanol extract | | | |  | Hot-water extract | | | |
| --- | --- | --- | --- | --- | --- | --- | --- | --- |
| Peak No. | RT (min) | RRT | UVW (nm) |  | Peak No. | RT (min) | RRT | UVW (nm) |
| 1 | 6.20 | 0.246 | 230 |  | 1 | 5.29 | 0.268 | 275 |
| 2 | 7.07 | 0.280 | 315 |  | 2 | 5.42 | 0.275 | 225 |
| 3 | 7.98 | 0.316 | 315 |  | 3 | 11.89 | 0.602 | 255 |
| 4 | 8.51 | 0.337 | 315 |  | 4 | 13.14 | 0.666 | 325 |
| 5 | 9.29 | 0.368 | 340 |  | 5 | 13.34 | 0.676 | 325 |
| 6 | 9.47 | 0.375 | 230 |  | 6 | 14.26 | 0.722 | 325 |
| 7 | 9.95 | 0.394 | 315 |  | 7 | 14.55 | 0.737 | 325 |
| 8 | 11.19 | 0.443 | 230 |  | 8 | 16.50 | 0.836 | 325 |
| 9 | 12.08 | 0.478 | 340 |  | 9 | 16.75 | 0.849 | 325 |
| 10 | 12.42 | 0.492 | 230 |  | 10 | 17.53 | 0.888 | 325 |
| 11 | 12.67 | 0.502 | 315 |  | 11 | 17.87 | 0.905 | 325 |
| 12 | 13.10 | 0.519 | 230 |  | 12 | 18.42 | 0.933 | 325 |
| 13 | 13.23 | 0.524 | 275 |  | 13 | 18.74 | 0.949 | 255 |
| 14 | 13.76 | 0.545 | 315 |  | 14 | 19.74 | 1.000 | 225 |
| 15 | 14.02 | 0.555 | 275 |  | 15 | 20.77 | 1.052 | 325 |
| 16 | 14.99 | 0.594 | 315 |  | 16 | 21.65 | 1.097 | 325 |
| 17 | 15.29 | 0.606 | 315 |  | 17 | 24.82 | 1.257 | 325 |
| 18 | 16.01 | 0.634 | 315 |  | 18 | 26.54 | 1.344 | 325 |
| 19 | 17.10 | 0.677 | 315 |  | 19 | 29.42 | 1.490 | 295 |
| 20 | 17.26 | 0.684 | 315 |  | 20 | 29.65 | 1.502 | 255 |
| 21 | 17.39 | 0.689 | 315 |  | 21 | 30.13 | 1.526 | 295 |
| 22 | 20.71 | 0.820 | 255 |  | 22 | 30.53 | 1.547 | 225 |
| 23 | 22.62 | 0.896 | 340 |  | 23 | 34.24 | 1.735 | 295 |
| 24 | 23.65 | 0.937 | 230 |  | 24 | 34.65 | 1.755 | 275 |
| 25 | 23.98 | 0.950 | 315 |  | 25 | 37.19 | 1.884 | 295 |
| 26 | 24.65 | 0.976 | 230 |  | 26 | 38.56 | 1.953 | 295 |
| 27 | 25.25 | 1.000 | 275 |  | 27 | 42.69 | 2.163 | 295 |
| 28 | 26.91 | 1.066 | 340 |  | 28 | 43.21 | 2.189 | 225 |
| 29 | 27.36 | 1.084 | 230 |  | 29 | 44.33 | 2.246 | 225 |
| 30 | 29.01 | 1.149 | 315 |  | 30 | 47.47 | 2.405 | 225 |
| 31 | 30.10 | 1.192 | 340 |  | 31 | 53.60 | 2.715 | 225 |
| 32 | 30.43 | 1.205 | 255 |  |  |  |  |  |
| 33 | 31.82 | 1.260 | 255 |  |  |  |  |  |
| 34 | 33.09 | 1.310 | 230 |  |  |  |  |  |
| 35 | 36.62 | 1.450 | 340 |  |  |  |  |  |
| 36 | 39.38 | 1.560 | 315 |  |  |  |  |  |
| 37 | 44.10 | 1.747 | 315 |  |  |  |  |  |
| 38 | 45.18 | 1.789 | 315 |  |  |  |  |  |
| 39 | 46.38 | 1.837 | 315 |  |  |  |  |  |
| 40 | 46.98 | 1.861 | 315 |  |  |  |  |  |
| 41 | 47.37 | 1.876 | 255 |  |  |  |  |  |
| 42 | 47.45 | 1.879 | 315 |  |  |  |  |  |
| 43 | 48.75 | 1.931 | 255 |  |  |  |  |  |
| 44 | 49.71 | 1.969 | 315 |  |  |  |  |  |
| 45 | 53.38 | 2.114 | 230 |  |  |  |  |  |

RT, retention time; RRT, relative retention time; UVW, ultraviolet absorption wavelength

Fig. S1.


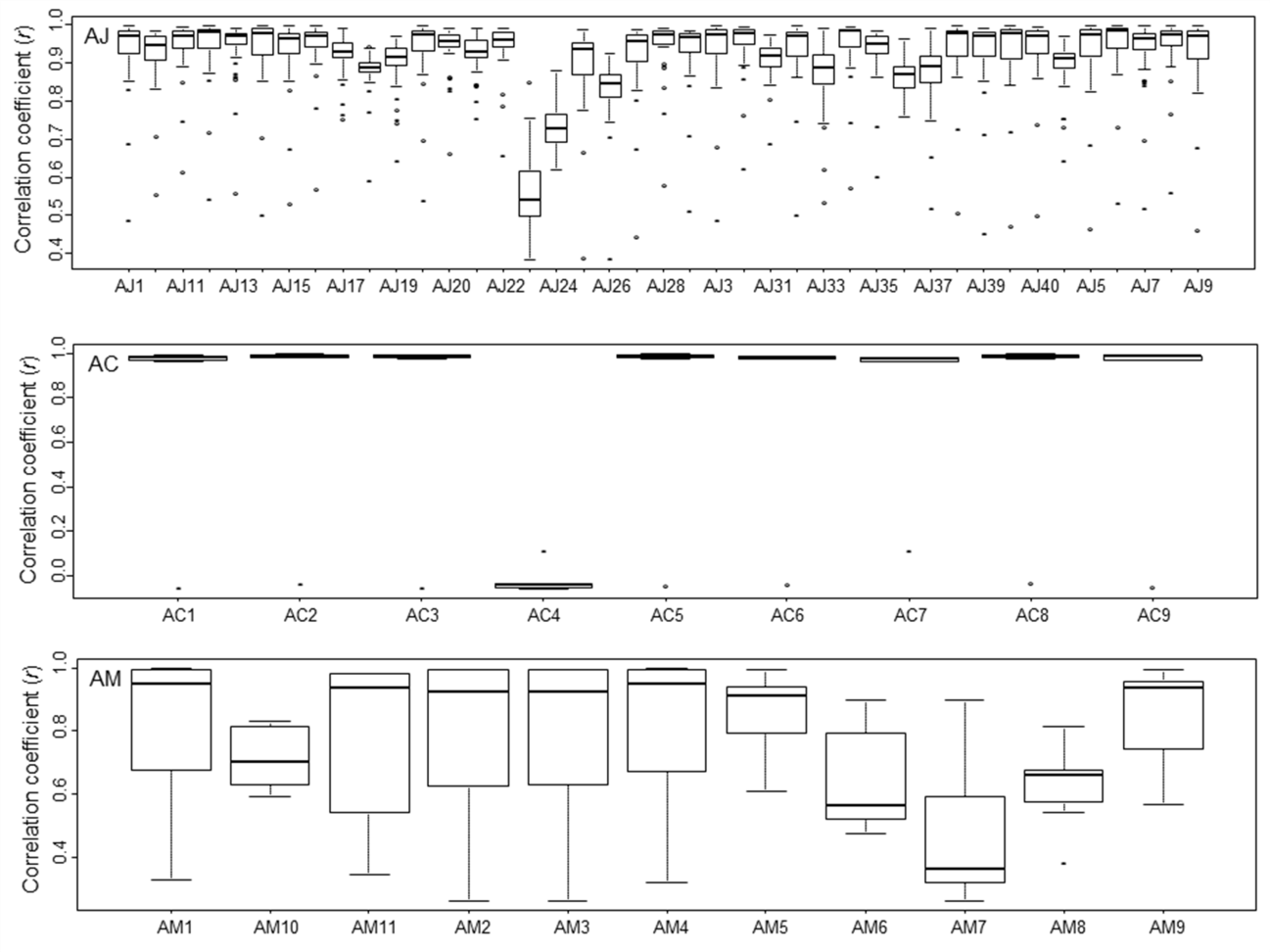


Fig. S2.


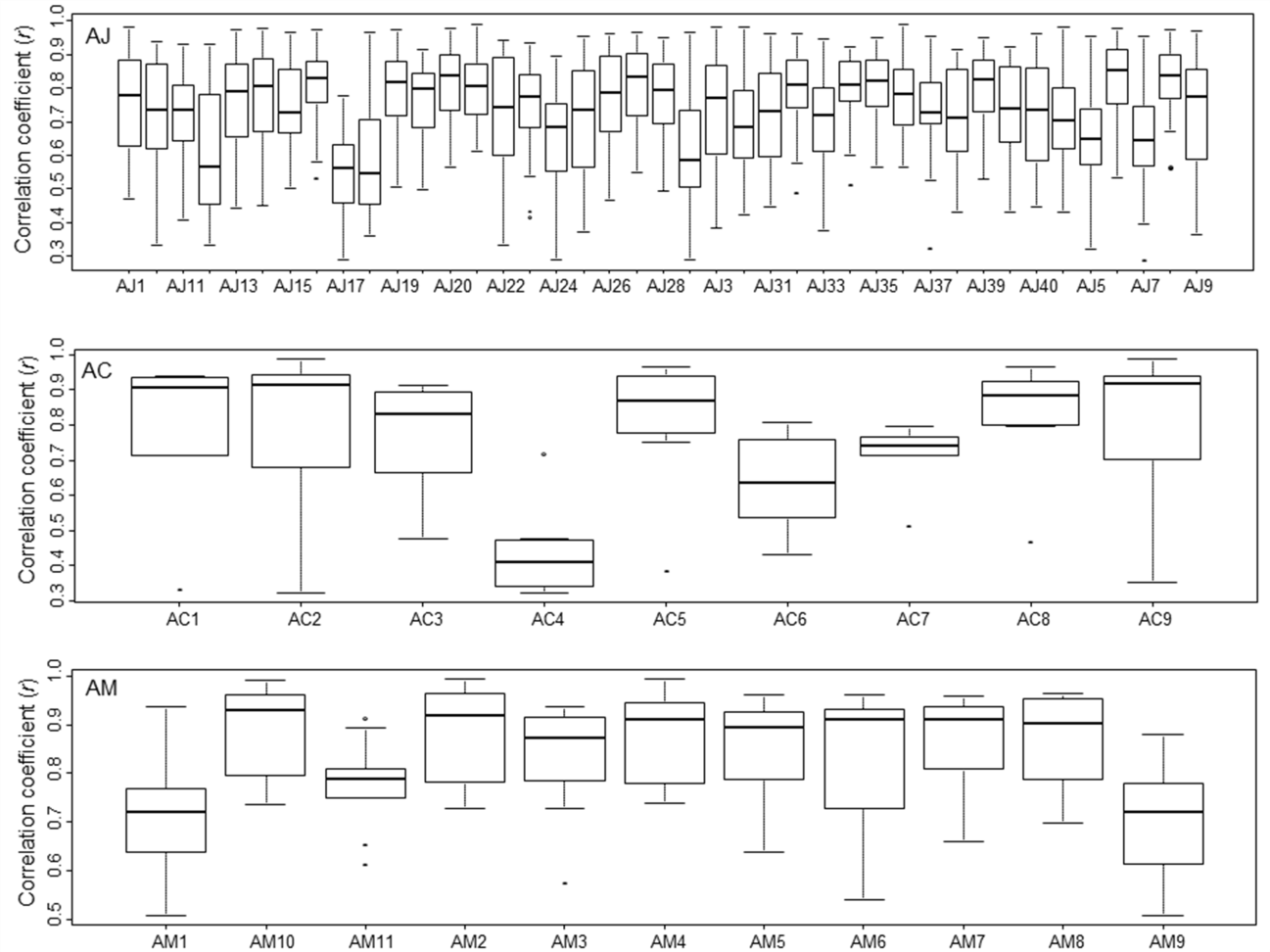

Supplement: Supplementary Materials — To read our article more intuitively, supplementary material is added as Table S1 and Figures S1 and S2. Table S1: peak number, retention time, relative retention time, and UV absorption wavelength of the reference peak in the chromatograms of the methanol extract and the hot-water extract. Figure S1: average coefficients of Pearson's correlation coefficient of Atractylodes samples from methanol extracts (AJ–AJ, AC–AC, and AM–AM). AC: A. chinensis Koidz.; AJ: A. japonica Koidz.; AM: A. macrocephala Koidz. Figure S2: average coefficients of Pearson's correlation coefficient of Atractylodes samples from hot-water extracts (AJ–AJ, AC–AC, and AM–AM). AC: A. chinensis Koidz.; AJ: A. japonica Koidz.; AM: A. macrocephala Koidz. [file 4860371.f1.docx]
